# Defining health-related quality of life for young wheelchair users: A qualitative health economics study

**DOI:** 10.1371/journal.pone.0179269

**Published:** 2017-06-15

**Authors:** Nathan Bray, Jane Noyes, Nigel Harris, Rhiannon Tudor Edwards

**Affiliations:** 1 Centre for Health Economics and Medicines Evaluation, Bangor University, Bangor, Gwynedd, United Kingdom; 2 School of Social Sciences, Bangor University, Bangor, Gwynedd, United Kingdom; 3 DesignAbility, Bath Institute of Medical Engineering, Bath, Somerset, United Kingdom; University of Illinois at Urbana-Champaign, UNITED STATES

## Abstract

**Background:**

Wheelchairs for children with impaired mobility provide health, developmental and psychosocial benefits, however there is limited understanding of how mobility aids affect the health-related quality of life of children with impaired mobility. Preference-based health-related quality of life outcome measures are used to calculate quality-adjusted life years; an important concept in health economics. The aim of this research was to understand how young wheelchair users and their parents define health-related quality of life in relation to mobility impairment and wheelchair use.

**Methods:**

The sampling frame was children with impaired mobility (≤18 years) who use a wheelchair and their parents. Data were collected through semi-structured face-to-face interviews conducted in participants’ homes. Qualitative framework analysis was used to analyse the interview transcripts. An *a priori* thematic coding framework was developed. Emerging codes were grouped into categories, and refined into analytical themes. The data were used to build an understanding of how children with impaired mobility define health-related quality of life in relation to mobility impairment, and to assess the applicability of two standard measures of health-related quality of life.

**Results:**

Eleven children with impaired mobility and 24 parents were interviewed across 27 interviews. Participants defined mobility-related quality of life through three distinct but interrelated concepts: 1) participation and positive experiences; 2) self-worth and feeling fulfilled; 3) health and functioning. A good degree of consensus was found between child and parent responses, although there was some evidence to suggest a shift in perception of mobility-related quality of life with child age.

**Conclusions:**

Young wheelchair users define health-related quality of life in a distinct way as a result of their mobility impairment and adaptation use. Generic, preference-based measures of health-related quality of life lack sensitivity in this population. Development of a mobility-related quality of life outcome measure for children is recommended.

## Introduction

### Prevalence of childhood disability and mobility impairment

Approximately 5% of children have some form of disability; globally 95 million children aged 14 or under are believed to have a disability [1]. In the United Kingdom (UK) 800,000 children and young people have a disability [2]. Mobility impairment is one of the leading causes of disability [3]. The National Health Service (NHS) is the largest supplier of wheelchairs and mobility aids in the UK, supporting over 1.2 million people with long-term mobility needs [4], around 70,000 of which are children [5].

Independent mobility offers a range of holistic benefits for children with impaired mobility, including functional mobility improvement [6], psychosocial development [7], increased independence [8] and reduced pain and deformity [9]. Appropriate wheelchair interventions are key to improving the outcomes of children with impaired mobility, including their health-related quality of life (HRQoL).

### Defining health-related quality of life

HRQoL is the perceived impact of health status on quality of life (QoL), including physical, psychological and social functioning [10]. Varni et al [11] found that, of the chronic conditions they evaluated, cerebral palsy had the most significant impact on the HRQoL of children. Vargus-Adams [12] and Dobhal et al [13] found that cerebral palsy predominantly impacts child HRQoL in the domains of physical function, physical independence, mobility and social integration. Wheelchair provision has been found to improve quality of life (QoL) [5,14] and more specifically HRQoL [15], however further research is required.

### Quality-adjusted life year framework

In the UK the National Institute for Health and Care Excellence (NICE) provides independent, evidence-based guidance to the NHS to inform healthcare funding allocation. At present there is limited robust economic evidence to inform NHS wheelchair provision and the design of NHS wheelchair services in an evidence-based manner [16]. Likewise, published evidence of paediatric wheelchair effectiveness tends to lack methodological quality [17].

Evidence-based decision-making requires robust evidence of cost-effectiveness to ensure that limited resources are used in a way that maximises potential benefits for patients [18]. NICE recommend the Quality-Adjusted Life Year (QALY) as a primary outcome measure in cost-effectiveness analysis [19]. QALYs represent an aggregate score of quantity and quality of life; they are calculated by multiplying the amount of time spent in a given health state by the relative societal preference for that health state. Due to their generic nature, QALYs can be used to compare disparate interventions and patient groups [20]. Health state preferences, or utilities, are derived from preference-based measures of HRQoL, therefore in order to calculate QALYs, robust preference-based HRQoL data is required.

### Preference-based health-related quality of life measurement

Preference-based approaches to HRQoL measurement are distinct as each combination of answers, representing a specific health state, is assigned a utility weight derived from the social desirability of that state, for instance ranging from death (0) to perfect health (1). Health state preferences are usually elicited from a large sample of the general population, and thus results reveal societal preferences for configurations of health. NICE stipulate that in the calculation of QALYs, preference weights must derive from a representative general population sample as societal resources should be allocated in a way that is relevant to the general population [21].

There is considerable debate regarding the use of patient or public preferences for health state valuation [22]. When assessing the desirability of hypothetical health states, individuals focus on the transition from their own health state to the hypothetical health state, and thus general population beliefs about the impact of disease and disability do not always reflect the lived experience [23,24]. When disability is presented to a member of the general public in the form of a hypothetical health state, the focus on personal transition means that processes of adaptation to health states are not accounted for, and thus there is a misunderstanding of the lived experience of disability [22].

Versteegh and Brouwer [22] state that differences in patient and public health state preferences reveal important information about perceived QoL. They conclude that both patient and general public preferences are valid sources of health state utilities, and therefore cost per QALY estimates should be calculated from the utility weights of both groups where possible.

### Applying methods of economic evaluation to wheelchair interventions

To date a small number of economic analyses of wheelchair interventions have been conducted [25–29], however these tend to lack methodological quality and nearly all focus on adults. Evidently there is a need for more high quality robust economic evidence, and appropriate data collection methods, to facilitate an evidence based approach to paediatric wheelchair provision. Some existing assistive technology outcome measures do approximate HRQoL measurement to a certain extent, for instance the Psychosocial Impact of Assistive Devices Scale (PIADS) has been found to be a robust psychosocial impact measure for assistive technology users [30], but it is neither preference-based nor directly measuring HRQoL constructs.

NICE and the QALY framework have become increasingly influential in UK healthcare. It is therefore important to generate data which adheres to NICE guidelines to ensure that it is comparable and informative in decisions about healthcare funding. This requires an understanding of how to apply methods of QALY calculation in specific contexts, such as paediatric wheelchair provision. This first requires an understanding of how young wheelchair users define HRQoL in relation to mobility impairment and wheelchair use (i.e. mobility-related QoL) in order to assess the appropriateness of NICE approved HRQoL measures, such as the EuroQoL Five Dimension (EQ-5D) [31].

Factors such as independence, socialisation, acceptance and integration are of particular importance to wheelchair users [8,14,32–34]. The ability of commonly used generic HRQoL outcome measures to accurately capture these domains in this specific population is unknown. There are currently no preference-based approaches to measuring HRQoL which have been developed specifically for use in mobility-impaired populations. Furthermore, generic preference-based measures appear to have limited sensitivity in the context of mobility impairment [35]. Previous researchers have resorted to amending the wording of the EQ-5D to increase relevance to people who use wheelchairs [36]; although pragmatic, this is not a valid use of such measures.

### Aim and research questions

The aim of this study was to determine how children with impaired mobility and their families define HRQoL and mobility-related QoL in relation to wheelchair use and mobility impairment. Furthermore, we sought to examine the applicability of standard HRQoL measures in this population, particularly those endorsed by NICE (i.e. EQ-5D) and designed specifically for use in children (i.e. Health Utilities Index (HUI) [37]). Research questions:

1. What are the key domains of HRQoL defined by children with impaired mobility and their parents, in relation to wheelchair use and mobility impairment?
2. To what extent do generic HRQoL measures reflect how children with impaired mobility and their parents define HRQoL in relation to wheelchair use and mobility impairment?

## Materials and methods

### Design and methodology

An exploratory descriptive study design was utilised. Data were collected through qualitative semi-structured interviews with young wheelchair users (≤18 years), their parent(s) or dyads of young wheelchair users and their parent(s). Qualitative Framework analysis was used to analyse the interview transcripts [38]. Qualitative Framework analysis is a popular approach in health-related research as it can be used to meet specific information needs [39]. Deductive *a priori* methods allows specific themes and issues to be examined in targeted populations using pre-specified aims and objectives [40]. In the context of health-related research this allows researchers to focus on a particular area of interest or phenomena, whilst maintaining systematic and transparent processes [41].

The study reported in this paper was part of a larger programme of research called the Wheels Project (funded by the National Institute for Social Care and Health Research PhD Studentship award), which also included a systematic review [16], quantitative assessment of HRQoL (paper in preparation) and a pilot discrete choice experiment questionnaire [42]. This paper presents only the qualitative findings.

This study was granted ethical approval by the North West Wales NHS research ethics committee (reference: 13/WA/0143) and an academic ethics committee at Bangor University.

For the purpose of this paper ‘child’ is defined as anyone aged 18 or under. The United Nations Convention on the Rights of the Child states that a child is any person under the age of 18 years [43]. Individuals aged up to 18 years were included in this research to be inclusive of individuals transitioning from child to adult wheelchair services. Although it is somewhat problematic to refer to teenagers and young adults as ‘children’, using a single term improves clarity within the paper. The term mobility-related QoL refers specifically to the impact of mobility impairment and wheelchair use on HRQoL.

### Data collection

Face-to-face semi-structured interviews were conducted in participants’ homes, guided by an interview schedule (S1 File). The interview schedule was piloted with a small sample (N = 10) of young wheelchair users (aged 11 to 18) at a children’s wheelchair charity beneficiary meeting in order to gauge their understanding of the questions and to refine the wording.

Each interview lasted for around an hour. The interview schedule was developed from a number of sources: the findings of a previous systematic review [16]; discussion within the research team; and with consideration of the EQ-5D-Y and HUI HRQoL measures. The questions facilitated participants to consider how they define HRQoL in relation to wheelchair use and mobility impairment, and to reflect on the ability of standard HRQoL measures to represent this definition. This included asking participants to discuss the domains and levels of the EQ-5D-Y and HUI measures in relation to their own definition of HRQoL. Parents were asked to discuss health and QoL in relation to their child.

The EQ-5D-Y and HUI measures were used to frame the interviews as they are two of the most commonly used generic preference-based HRQoL measures. The EQ-5D is recommended by NICE for use in cost-effectiveness analysis [44], and the HUI was the only preference-based HRQoL measure designed specifically for use in children and adolescents at the time of designing the study.

The EQ-5D-Y measures HRQoL using five domains: mobility; self-care; usual activities; pain and discomfort; and anxiety and depression [31]. The EQ-5D was originally developed for use in adult populations. A child version, the EQ-5D-Y, has been validated for proxy reporting from age four [45] and child self-reporting from age eight [46]. EQ-5D respondents are asked to rate their health on each domain using one of three possible responses: no problems, some problems, a lot of problems. A 5-level version of the EQ-5D has been validated in adults, but not children [47].

The HUI contains the HUI2 and HUI3 systems [37]. It comprises a 15-question self-completion questionnaire, with each question presenting between four and six possible responses (i.e. levels). The HUI covers a range of HRQoL domains, including: sensation, speech, mobility, dexterity, emotion, cognition, self-care and pain. It is validated for proxy use from age five, and child self-reporting from age eight [37].

### Recruitment and sampling

The sampling frame was young wheelchair users (≤18 years) with long term mobility impairments and their parent(s). The sample was stratified by the age of the child (0–5 years; 6–15 years; 16–18 years) and interviewee structure (child; parent; child/parent dyad). Potential participants were sent postal information about the study and indicated their consent to participate by returning a completed demographics questionnaire (which contained an initial consent/assent form). A date and time for a face-to-face interview was then arranged via a telephone follow-up. Participants were recruited between June and October 2013 from three recruitment sites: an NHS wheelchair service, a charity powered wheelchair (PWC) manufacturer/supplier and a children’s wheelchair charity.

Before beginning the interview the study was explained in full to participants. Participants completed a second consent/assent form to indicate that they understood the purpose of the study and agreed to take part in the interview. Children under the age of 16 completed an assent form and their parents completed a proxy consent form.

### Response rate and sample size

A total of 125 study packs were distributed across England and Wales by the three recruitment sites. 38 initial HRQoL/demographic questionnaires were returned (initial response rate of 30.4%). Of the 38 child/parent dyads invited to take part in the interview ten declined. 27 interviews were conducted (one of which contained two child participants from the same family), giving a secondary response rate of 73.7%. An overall interview response rate of 22.4% [N = 28] was observed for all of the 125 invitation packs sent out.

In total 17 parents decided to take part on their own, either because their child was too young to participate [N = 12; all under 5's] or because they felt that it was not suitable for their child [N = 5; all 6 to 15 year olds]. Four children over the age of 16 took part on their own and seven child/parent dyads took part in the interview together. In total 24 parents and 11 children were interviewed. Full disclosure of demographic details are presented in Tables 1 and 2.

**Table 1:** Child demographic characteristics (child, parent and dyad samples).

Demographic characteristics	Children N = 4 (%)	Parents N = 17 (%)	Dyads N = 7 (%)
Child gender			
Female	1 (25)	6 (35.3)	3 (42.9)
Male	3 (75)	11 (64.7)	4 (57.1)
Child age			
5 years or under		12 (70.6)	
6–15 years		5 (29.4)	4 (57.1)
16–18 years	4 (100)		3 (42.9)
Child ethnicity			
White British	4 (100)	16 (94.1)	7 (100.0)
Other mixed background		1 (5.9)	
Child diagnosis			
Porencephaly		1 (5.9)	
Cerebral Palsy	3 (75)	11 (64.7)	6 (85.7)
Muscular Dystrophy	1 (25)	2 (11.8)	
Rett syndrome		1 (5.9)	
Lissencephally		1 (5.9)	
Chromosome deletion		1 (5.9)	
Hemiplegia / stroke			1 (14.3)
Child Frequency of equipment use			
A little of the time		1 (5.9)	
Some of the time		4 (23.5)	
Most of the time		3 (17.6)	1 (14.3)
All of the time	4 (100)	9 (52.9)	6 (85.7)
Child Type of equipment used			
Powered		2 (11.8)	
Manual	1 (25)	7 (41.2)	3 (42.9)
Manual and powered	3 (75)	8 (47.1)	4 (57.1)

**Table 2:** Parent sample demographic characteristics.

Demographic characteristics	Parents N = 17 (%)	Dyads N = 7 (%)
Parent gender		
Female	15 (88.2)	7 (100.0)
Male	2 (11.8)	
Parent age		
21–29 years	2 (11.8)	
30–39 years	11 (64.7)	1 (14.3)
40–49 years	4 (23.5)	5 (71.4)
50–59 years		1 (14.3)
Parent ethnicity		
White British	16 (94.1)	7 (100.0)
White & Asian	1 (5.9)	
Parent marital status		
Married	11 (64.7)	7 (100.0)
Co-habiting	3 (17.6)	
Single	2 (11.8)	
Separated	1 (5.9)	
Annual household Income		
£5000–15,000	3 (17.6)	
£16,000-£25,000	3 (17.6)	
£26,000-£35,000	2 (11.8)	1 (14.3)
£36,000-£50,000	5 (29.4)	4 (57.1)
£51,000-£75,000	2 (11.8)	2 (28.6)
£75,000 or more	2 (11.8)	
Parent employment status		
Full-time	3 (17.6)	
Part-time	6 (35.3)	3 (42.9)
Unemployed / stay at home parent	8 (47.1)	4 (57.1)

### Data handling and analysis

Each interview was transcribed verbatim. Identifiable data were deleted from the transcripts to maintain confidentiality. Transcripts were not returned to participants for comments or corrections due to time constraints. The software nVivo v9.2 was used for qualitative data handling.

An *a priori* thematic coding framework (S2 File) was used to line-by-line code the transcripts, and was developed from a number of sources: a previous systematic review [16]; the interview schedule; familiarisation with the interview transcripts; research team discussion; and the HRQoL domains of the EQ-5D-Y and HUI measures. Inductive codes which arose during the coding process were added to the coding framework. Codes were grouped into categories of related codes, which were subsequently refined into higher order analytical themes giving a broader understanding of the coded transcripts and the relationship between categories of codes.

Child and parent responses were analysed separately to account for their different but equally valid perspectives. Child age was also considered in the analyses, with separate analysis age groups defined as under 5’s, 6 to 15 year olds and 16 to 18 year olds to represent stratification of NHS wheelchair provision. Type of wheelchair used was initially used to group data, but was later rejected due to consensus across the groupings.

Once coded, charts and maps were used to integrate the data to gain a richer understanding of the phenomena. This included integration of child and parent responses. Data were then used to build an understanding of how children with impaired mobility and their parents define mobility-related QoL, and the subsequent applicability of standard measures of HRQoL.

### Theoretical position

We approached this research from an extra-welfarist, societal and social model of disability perspective, underpinned by principles of disability equality and utility theory.

The social model of disability postulates that people with impairments become disabled as a result of social oppression and discrimination. Disability is therefore defined as being a direct result of societal barriers to participation and independence imposed on people with impairments [48]. This theoretical position necessitated a broader view of disability and impairment in this study, looking beyond traditional medical definitions of health and disability. Thus, we focussed on a wide range of topics in the interviews and analyses.

Utility theory underpins popular methods of economic evaluation in healthcare, such as QALYs. Although utility cannot be derived directly, it is possible to rank different health states in order of societal preference. Therefore, the acceptable cost of a healthcare service or intervention is related to the marginal utility gains it provides.

Extra-welfarism is a normative approach to the analysis of health and healthcare, which includes outcomes such as individual utility, happiness, social interaction and pain. This approach is relevant to health as the development of public healthcare systems reflects the need to allocate resources fairly and efficiently within financial constraints. The QALY approach has a basis in extra-welfarism.

Utility theory and extra-welfarism underpin the QALY framework, therefore because we focused on the use of HRQoL data for economic evaluation purposes, these theories informed our understanding of HRQoL and the data we sought to collect.

The study reported in this paper was underpinned by an overarching conceptual framework developed as part of a previous systematic review [16]. This conceptual framework maps areas for future research and service development to facilitate cost-effective wheelchair services for children with impaired mobility. One of the key areas identified for development was better outcome measurement, specifically relating to non-clinical needs, such as HRQoL.

### Qualitative research reporting standards

To acknowledge the importance of explicit and comprehensive reporting of qualitative research, this paper follows the COREQ checklist for qualitative reporting standards [49]. As primary researcher, Dr Nathan Bray was solely responsible for conducting, coding and analysing all interviews. Wider discussion of the data within the research team was used to shape and test interpretations and to ensure internal validity. All participants were unknown to the research team prior to conducting the interviews.

## Results

Participant quotes are presented as informative and clear representations of specific analytical themes. Irrelevant information has been replaced with ellipses […] to facilitate ease of reading. Repetitive speech and linguistic fillers (such as ‘um’) have been removed. Where there is more than one respondent presented in a single quote the following tags have been used for clarity: ‘R:’ for researcher, ‘C:’ for child, ‘M:’ for mother and ‘F:’ for father. The term child is used to refer to children with impaired mobility aged 18 or under who use a wheelchair. The term QoL is used to refer to mobility-related QoL; a specification of HRQoL. Participant ID numbers are presented so that multiple quotes from the same individual can be identified.

### Defining mobility-related QoL

**Fig 1:**
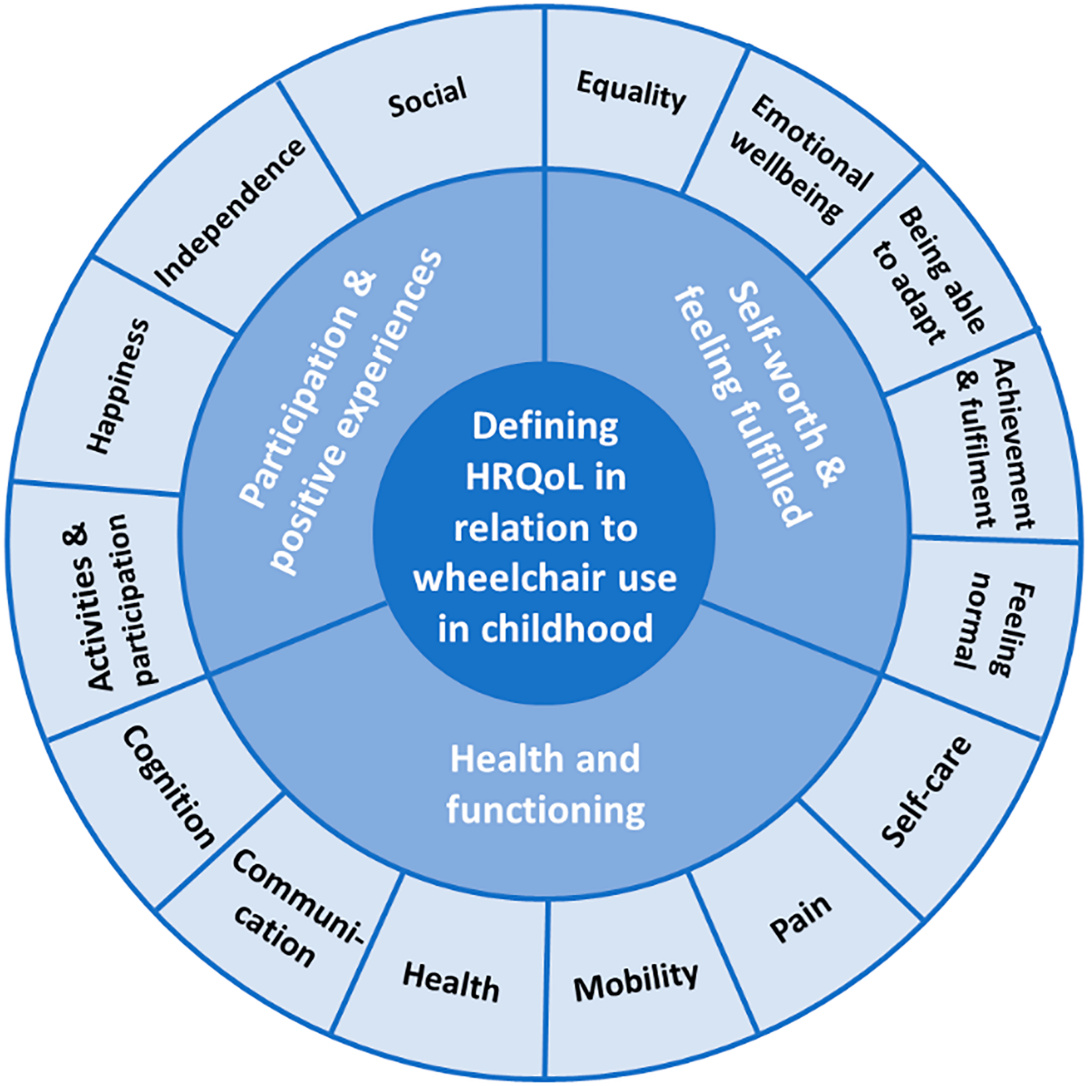
Defining mobility-related QoL in childhood. A thematic summary and map of mobility-related QoL domains for children and young people.

In total, 15 categories were used by participants to define mobility-related QoL. The most commonly identified code categories were independence, social interaction and activities/participation. The 15 categories were synthesised to form 3 analytical themes, these were: participation and positive experiences; self-worth and feeling fulfilled; health and functioning. See Fig 1 for a breakdown of categories and analytical themes.

#### Participation and positive experiences

Being able to take part in positive and enjoyable experiences was an important aspect of defining QoL for children and parents across all age ranges. Furthermore, the categories of independence and activities/participation were the only common categories found across all sub-groups (i.e. 3 child age groups; children and parents). A key aspect of participation for children and parents alike was being able to take part in activities that are meaningful to the child, without restriction.

Mother of 4 year old male (P21): *R*: *What does the term “QoL” mean to you in relation to [CHILD’S NAME] and other children with disabilities*?

*M: Just that they get to have the opportunities and enjoyment of other children, really. And that what he is doing is meaningful to him*.

Adaptations and equipment such as wheelchairs played a vital role in removing barriers to participation and providing alternative ways for children to take part in activities. However, structural and environmental barriers, such as inadequate facilities or poor access for wheelchairs, were seen as restrictive.

Mother of 10 year old female (P28): *QoL is them being able to do anything they want to be able to do*. *And not being prevented from doing that by something stupid like not being able to access it because there’s no ramp*. *Or no lift or something like that*.

Closely related to activities and participation was social interaction. The ability to interact with friends and participate in social situations was of particular importance to older children. Parents did not place as great an emphasis on social interaction, and tended not to differentiate this from other forms of activities and participation. The importance of independence underpins these key aspects of QoL. Children and parents described how wheelchairs and other adaptations positively impact independence by providing freedom of movement and the ability for children to make their own decisions.

17 year old female (C29): *Everywhere I went I was pushed around in this buggy when I went out*. *I was like 10 [years old] in a buggy*, *if you know what I mean*. *I felt left out quite a lot*, *there's just no independence just sitting in a buggy*. *I think it's really good that little ones can have [PWCs] younger*.

Older children discussed taking control of their own lives and being in charge of their own decisions. For many children independence was about being able to socialise and to do activities that were important to them, without feeling like they were restricted by their mobility impairment. Although self-sufficiency was important, it was accepted that assistance would be required. Importantly, children wanted to feel in control of how assistance and support was provided to them.

17 year old male (C24): *A good QoL for me I think is being*, *not necessarily being entirely independent*, *but being in charge of my own doings*, *so to some extent that means I’m able to move myself about in my chair but in other respects where I require assistance*, *like I have an assistant in college but I’m still in charge of asking him for help*, *asking him to get my books out*. *I am in charge of my own life*, *I’m not being dictated to by other people or really by my disability*.

#### Self-worth and feeling fulfilled

The analytical theme of self-worth and feeling fulfilled was expressed through a number of categories by both children and parents. Self-worth was defined in a number of ways by children, including the ability to set and achieve goals; feeling equal and free from prejudice; being able to adapt and feel normal; and good emotional wellbeing.

For older children achievement was a key attribute in defining QoL, specifically being able to set and achieve goals. This category linked with independence and autonomy, as children indicated that being able to set personal goals allowed them to express themselves and focus on what was important to them. Achievement was described as being able to make the most of opportunities, regardless of perceived barriers to access or participation.

18 year old male (C20): *It’s also trying to give yourself as many opportunities as you can and taking advantage of them because there’s certain opportunities that you might only get once*. *Take advantage of it*. *To be honest when I was a bit younger some of the things I was able to do I can’t do now but I’m pleased that actually I did do them because I’ve enjoyed doing them and at least I know I’ve had that experience*.

Emotional wellbeing was a common theme for parents of young children. They expressed that QoL was about children having a happy and enjoyable childhood free from worry.

Mother of 2 year old female (P06): *[QoL is] a child being a child and just not having to worry about*, *not that she will be at this age*, *but not having to worry about how they look or feel*. *She's just enjoying being a child because she can move around*.

Older children and their parents did not discuss emotional wellbeing directly in great detail, however, they did refer to coping with disability. For many of the older children and their parents adaptations such as wheelchairs were seen as an important tool in the process of coming to terms with disability and impairment. Ability to cope with and accept a disability was to some extent seen as defining how limitations would affect QoL.

17 year old male (C25): *Disability is something that you cannot get away from*, *and if you weren’t able to deal with that fact you would quickly become very depressed*, *very helpless and you'd quickly think “my life isn’t worth living*.*”*

Parents of young children identified that QoL was in part about their child having equal opportunities to participate and not being excluded from anything, which linked with being able to participate and enjoy activities.

Mother of 3 year old male (P04): *[QoL is] that he’s given every opportunity to enable him to do his best*, *really*. *And making sure that he gets the opportunity and that when he gets it he can do it as well*, *whatever it is it’s been adapted so that he can do it*.

Older children stated that they wanted to be treated the same as other children and didn’t want to be stereotyped or discriminated against due to their disability.

17 year old male (C25): *One of the big things for me is not being stereotyped*, *not being discriminated against*, *not having to fight for things*, *I sometimes feel really under pressure from people*.

#### Health and functioning

Children and parents across all age groups stated that with correct adaptations the QoL of a child with impaired mobility can be equivalent to that of an able-bodied child. This requires correct provision of adaptations which promote the best functional ability for that child, not necessarily working towards ‘normal’ functioning.

16 year old male (C16): *I've had conversations with friends who are completely able-bodied and they've sort of said “Do you wish you could do some of the things I can do*?*” but I said “But do you wish you could put a basketball ball in the basket like I can*?*” It's like*, *everybody's got things they can do better than other people*. *It's like*, *they can walk better than I can but I can push a wheelchair better than they can*.

Traditional definitions of functional abilities, such as walking and self-care, were not widely used to define QoL. Self-care was only discussed by one child and the relative importance of functional mobility (i.e. walking) was only discussed by two parents of children under the age of 5. They stated that walking and freedom of movement without adaptations are basic needs which can affect QoL when restricted, although this was not a widely stated opinion.

Mother of 4 year old male (P26): *People think “well he’s never done it*, *he’s never walked*, *he won’t miss it*.*” It’s not true*, *you have a basic need to get up on your feet and walk from being 6months old*, *you have that*, *you need to do it…You can’t do anything*, *you can’t go anywhere that you want to go*, *you know*. *You want to go and get a drink*, *you want to pick that toy up*, *you want to go over to your friends*.

All subgroups discussed the beneficial effects of adaptations, specifically the role of wheelchairs in raising QoL; wheelchairs were identified as being an intrinsic part of defining QoL.

Mother of 5 year old male (P08): *For people with disabilities*, *QoL does come down to your adaptations and how you adjust your life to make it good quality*.

Participants remarked that there is a balancing act between maintaining normal functioning and deciding to use adaptations. For instance, a child may physically be able to continue walking but may experience more pain doing so. It is therefore counterproductive to assume that QoL is defined by ‘normal’ functioning in this situation, as QoL may be maximised by reducing that ‘normal’ functioning. In this respect, defining QoL is about maximising functioning regardless of ability or the requirement for adaptations.

17 year old male (C24): *There’s a tipping point where you go “It’s not worth [walking] anymore*, *it hurts too much*, *it’s not practical*, *I’m too tired”*.

Health and pain were used to define QoL by parents of younger children but were seen as secondary to other areas of QoL, and separate to disability. This could be due to the implicit importance of good health and being free from pain.

Mother of 2 year old male (P01): *I think [QoL is] just being well*, *because we’ve had a lot of problems with various things*. *So his health*, *at the minute*, *is the main thing*. *Just to see that he’s not in pain*. *He has quite bad seizures which are controlled at the minute*.

It is of note that across all sub-groups participants felt that disabilities acquired later in life would have a greater impact on HRQoL than congenital mobility impairments. The loss of abilities and functioning was perceived to be more detrimental to HRQoL than having never experienced them.

17 year old male (C25): *When you have [a disability] from birth you almost don’t have to deal with it*, *because you know*…*you don’t know any different*, *and when you do start knowing any different you deal with that again*. *The difference is your Mum and Dad are there to feed you*, *make sure you're alright*. *When a disability happens when your parents are not around*, *or when you've not had it from [birth]*…*you kind of know what you’ve lost*.

### Relevance of generic measures of HRQoL: EQ-5D-Y and HUI

Participants were asked to review the content validity of the EQ-5D-Y and HUI measures and to discuss the relevance of each HRQoL domain to them (or their child). The general consensus was that the domains of HRQoL on these measures were relevant to some extent. However, often the available responses for each question were insufficient or the question wording was not sensitive enough for their abilities, particularly the mobility/ambulation domains. For instance, participants stated that although issues of ambulation, dexterity and impaired senses can impact HRQoL, these issues can essentially by negated by appropriate adaptations. Furthermore, all participants indicated that although mobility can impact HRQoL, mobility should not just be defined as walking.

17 year old male (C24): *I think as far as the five [domains of HRQoL on the EQ-5D] are concerned*, *they kind of cover the broadest range*, *but the actual three answers that you’re given for each one kind of…it was difficult for me to fill it out*. *I find these things very difficult because you try and be extremely positive as possible about things*, *and there seem to be gaps almost where I fit*. *I found it difficult to fill out just because you think “I don’t fit here*, *or I don’t fit here”*.

The 15 categories used to define mobility-related QoL were mapped on to the HRQoL domains of the EQ-5D and HUI, see Table 3. The results indicate that the EQ-5D and HUI measures do not cover a range of non-health HRQoL domains which were of importance to participants in this study. This is most apparent for the participation and positive experiences analytical theme, as there is no differentiation between different types of ‘usual activities’ on the EQ-5D, and no consideration of participation and positive experiences on the HUI. Given that participants also raised concerns about the wording of these measures and the available responses, it is clear that the EQ-5D and HUI measures are not fully representative of how young wheelchair users and their parents in this cohort defined mobility-related QoL.

**Table 3:** Comparing domains of mobility-related QoL with HRQoL domains of the EQ-5D-Y and HUI.

Mobility-related QoL analytical themes	Mobility-related QoL code categories	EQ-5D-Y HRQoL Domains	HUI 2 and 3 HRQoL Domains
Participation and positive experiences	Activities/participation	Usual activities	-
	Happiness	-	-
	Independence	-	-
	Social	-	-
Self-worth and feeling fulfilled	Achievement/fulfilment	-	-
	Being able to adapt	-	-
	Emotional wellbeing	Anxiety/depression	Emotion
	Equality	-	-
	Feeling normal	-	-
Health and functioning	Cognition	-	Cognition
	Communication	-	Communication
	Health	-	-
	Mobility	Mobility	Ambulation/mobility
	Pain	Pain/discomfort	Pain
	Self-care	Self-care	Self-care
Others:			1. Senses
			2. Dexterity

## Discussion

Participants defined mobility-related QoL through three distinct but interrelated concepts: participation and positive experiences; self-worth and feeling fulfilled; and health and functioning. Children and parents showed general consensus, however parents of younger children placed greater emphasis on health and functioning. The domains of HRQoL defined by standard HRQoL measures (such as the EQ-5D-Y and HUI) are to some extent applicable to children with mobility impairments, however important considerations of participation, positive experiences and self-worth related to mobility-impairment are not accounted for. Furthermore available responses on these measures are not sufficiently sensitive in this context due to a focus on normative functioning.

Generic preference-based HRQoL measures are scored using health state preferences derived from the general public, which may be problematic in this context as the preference of an able-bodied person for a state of immobility is almost certainly going to be low. However, as stated by participants in this study and others, inability to walk does not necessarily discount HRQoL [50,51]. This is a key distinction to make when considering the use of general population value sets in disabled populations: preferences will always be for a state of ability rather than disability. Taking into account the social model of disability, it is fundamentally wrong to apply an able-bodied perspective to a disabled phenomenon.

Commonly used preference-based HRQoL measures, such as the EQ-5D-Y and HUI, are potentially not fit for purpose in this population, as subsequent utility scores reflect a general population perception of HRQoL rather than the perceptions of people living with mobility impairment. Although this is representative of the wider societal view of the health states, it is not representative of the population that was intended to be studied. Perhaps the solution is to think beyond generic QALYs and clinical effectiveness, and to consider mobility specific approaches to HRQoL measurement, or to use relevant patient preferences for health states [22,52].

In circumstances where generic measures lack sensitivity, condition-specific approaches to QALY calculation can be used. Preference-based condition-specific HRQoL measures have been developed successfully in areas such as visual impairment [53], dementia [54] and epilepsy [55]. The results presented in this paper support the need to develop a preference-based mobility-related QoL outcome measure which could be used to accurately measure utility gains associated with mobility interventions in disabled populations, for both children and adults.

One of the key findings was the importance of independence in defining and enhancing HRQoL. Wheelchair provision should enable and sustain independence [56], and promote physical, cognitive and social development [57]. Without the ability to move independently children are unable to have full control over their social interactions and their ability to participate in activities. Furthermore, without appropriate access to enable independent movement children are restricted in their ability to socialise and participate. Children and parents seek an appropriate level of independence relative to the child’s abilities, and this relates to removing barriers that oppose independence. Wiart et al [8] found that mothers believed that wheelchairs enabled their child to take part in age-appropriate activities and reduced their need for assistance. Likewise, Evans et al [34] found that after provision of a PWC children experienced increased independence, which led to increased socialisation and participation.

Traditional functional abilities such as walking and basic self-care were not widely used to define mobility-related QoL. Instead participants focussed on mobility by other means (such as wheelchairs) and ability to adapt. This indicates that positive abilities and functioning are much broader than traditional definitions, such as simply being able to walk. Several participants stated that children with impaired mobility can achieve a QoL on par with an able-bodied child if the correct adaptations are in place to facilitate this, although this could not be replicated on the either the EQ-5D-Y or HUI measures using their current descriptive systems.

Children and parents of older children used terms such as happiness and emotional wellbeing less frequently than parents of younger children. This suggests a difference in QoL perspectives associated with age, as nebulous terms such as happiness become replaced with specific activities or functioning which provide happiness and wellbeing. The role of health and function in defining QoL was of key importance to parents of younger children, but was less important for older children and their parents. This reflects previous evidence which found that parents of children with impaired mobility go through a process of learning to accept the changing mobility and equipment needs of their child [8].

The EQ-5D was not originally designed for use in children, and although the youth version has been validated in younger age groups, the domains and levels of the descriptive system were not designed specifically around the testimony of children, let alone people with disabilities. Child-specific measures of HRQoL, such as the CHU-9D, [58] are now available, although additional research is needed to verify their applicability for children with disabilities more generally.

An important consideration is whether generic measures are appropriate in populations where health and QoL is likely to fluctuate or degenerate rapidly. For instance, if HRQoL fluctuates on a day-to-day basis due to changing functional abilities, or if health decreases rapidly in a short space of time, then basing HRQoL estimates on a single time point could cause validity and reliability issues. Likewise, if regular retests were used to counter this then the measure would need to be highly sensitive to change over time. HRQoL measures tend to be comprised of relatively simple descriptive systems, which may be insufficiently sensitive to detect rapid change. In this respect a patient-generated outcome measure could prove to be more efficient. From a clinical perspective this would be an informative approach to measuring effect, however from a health economics perspective patient-generated outcomes lack genericity for utility measurement. Each individual would have very specific needs and priorities, thus making comparisons between people and groups difficult. Furthermore, it would not be possible to generate a generic health state preference for every possible scenario. Likewise, identifying preferences at the individual level would prove too time consuming. Generic or condition-specific measures with higher sensitivity are required, for instance a preference based mobility-related QoL outcome measure developed through consultation with people with mobility impairments and validated in children.

There was general consensus that disabilities acquired later in life would have a greater impact on HRQoL than congenital mobility impairments. This correlates with the perception that HRQoL is defined by personal ability, and thus a change in that ability detrimentally impacts HRQoL. The important factor is thus the baseline level of ability and the ability to cope with subsequent change. Focusing on ‘normal’ functioning can potentially detrimentally impact wheelchair users, as their HRQoL may in fact be increased by reducing walking and favouring wheelchair mobility due to pain related to walking. This reflects Sen’s theory of capability, which differentiates achieved functionings and capabilities; focussing simply on achieved functioning presupposes optimal capabilities [59].

### Strengths and limitations

A quarter of the interviews were conducted with the parent and child present at the same time, which may have influenced both parent and child answers. Most participating children were aged 16 or over (72.7%) and thus this study potentially misses the perspectives of younger wheelchair users. There were inherent problems with including young children in the interviews due to parental concern, child ability and communication skills. Future research would benefit from focusing on younger children and utilising a greater array of child friendly research methods to elicit responses.

It is important to consider the transferability, credibility, confirmability and dependability of the results [60]. Due to the study demographics and the relatively small sample size it is difficult to judge whether these results are transferable to other comparative settings. The sample is potentially not representative of the wider population of young wheelchair users, as the demographics show over-representation of children with cerebral palsy from relatively high-income families. We have provided a clear and thorough breakdown of participant demographics to give specific context for these results.

The analytical themes generated from the interviews are reflective of the opinions expressed by participants, highlighted by the illustrative quotes throughout this paper. The interview schedule was reviewed after each interview so that we could be reflexive to new and emerging themes; only minor amendments were required in practice. Due to time constraints participants were not involved in analysis and did not have the opportunity to verify transcripts, which potentially may have affected the credibility of the findings. However, all interviews were transcribed verbatim and double-checked for errors prior to formal analysis. The use of Framework Analysis allowed for a transparent and flexible approach to the data analysis, however, the *a priori* nature of the framework likely produced different results than a more inductive process might have.

It is good practice for multiple researchers to code interview transcripts. However, as this research was carried out as part of a PhD studentship, funding and time was limited. Therefore, coding was only carried out by one researcher. To increase consistency and maintain validity, the data were widely discussed within the wider research team to help shape interpretations and analyses.

## Conclusions

Young wheelchair users and their parents defined mobility-related QoL through three distinct but interrelated analytical themes: participation and positive experiences; self-worth and feeling fulfilled; and health and functioning. For the purpose of economic evaluation it appears that generic preference-based measures of HRQoL lack sensitivity to elicit reliable utility data from children with mobility impairments. Future economic research in this field must consider alternative approaches to utility measurement. Child-specific preference-based utility should be used as a basis for cost-utility analysis at present. This requires additional research to understand the applicability of measures such as the CHU-9D in disabled populations, and potentially the development of mobility-related utility measures.

## Supporting information

S1 File Interview schedule. Interview schedule and questrions used to guide semi-structured interviews with children and parents.
(PDF)

S2 File *A priori* thematic coding framework. *A priori* thematic coding framework used to code transcripts.
(PDF)

## Abbreviations

CHU-9D: Child health utility nine dimension
COREQ: Consolidated criteria for reporting qualitative research
EQ-5D: EuroQoL five dimension
EQ-5D-Y: EuroQoL five dimension youth version
HRQoL: Health-related quality of life
HUI: Health utilities index
NHS: National Health Service
NICE: National Institute for Health and Care Excellence
PIADS: Psychosocial Impact of Assistive Devices Scale
PWC: Powered wheelchair
QALY: Quality-adjusted life year
QoL: Quality of life
UK: United Kingdom
