# Supplementary material for: Defining health-related quality of life for young wheelchair users: A qualitative health economics study
Source: PLoS One. 2017 Jun 15;12(6):e0179269. doi: 10.1371/journal.pone.0179269 (PMC5472290; doi:10.1371/journal.pone.0179269)
Supplement: S1 File — Interview schedule and questrions used to guide semi-structured interviews with children and parents. (PDF) [file pone.0179269.s001.pdf]

Interview schedule

Questions are not restrictive, discussion around topics encouraged.

(amend wording for parents e.g. your = your child's)

Overall topics

Wheelchair services- assessment, waiting times, quality of equipment

Wheelchairs- good/bad aspects, affect on abilities/activities

Quality of life – impacts from wheelchair use, defining quality of life, outcome measure suitability

Wheelchair questions

1. How long have you been using a wheelchair?
2. Are you able to get around without a wheelchair?
3. Please describe your experience of getting a wheelchair
    a. Who supplied your wheelchair?
    b. How long did it take to get your wheelchair?
    c. Does your wheelchair meet your expectations?
4. Please describe when and where you use your wheelchair
    a. Do you have more than one wheelchair?
5. How does your wheelchair positively affect your life?
6. How does your wheelchair negatively affect your life?
7. Please describe or draw your perfect wheelchair
8. Please describe some situations when you weren't able to use your wheelchair
9. What is your experience of wheelchair services?
10. What would you change about wheelchair services?
11. PARENTS / YOUNG PEOPLE Would you consider buying a wheelchair if you couldn't get what you wanted from a wheelchair service?
    a. How much would you be willing to spend?
12. When you were assessed for your wheelchair, what was the most important part of the assessment for you?
13. Were all of your needs assessed and catered for when you were supplied a wheelchair?
    a. Please describe how wheelchair assessments could be improved
14. To what extent has your wheelchair helped you to access education or work?

Quality of life questions

1. What does the term ‘quality of life’ mean to you?
2. Please describe your quality of life
    a. Which aspects of your life have the biggest impact on your quality of life, such as ability to get around, socialising with friends or managing pain and discomfort
3. How does your wheelchair affect your quality of life?
4. What changes to your wheelchair would improve your quality of life?
5. Please review these questionnaires [health-related quality of life measures]
    a. How relevant are the questions to you?
    b. The EQ-5D defines quality of life in 5 ways- mobility, looking after yourself, doing usual activities, pain/discomfort and feeling worried, sad or unhappy. If you had to define quality of life what would be the 5 most important aspects of quality of life?
    c. To what extent do these questionnaires represent your understanding of the term quality of life?
6. Do you think your quality of life would be worse if you didn’t have a wheelchair / didn’t have a wheelchair you could control yourself?
7. What activities does your wheelchair help you to do?
8. To what extent has your wheelchair improved the quality of life of your family?
