# Supplementary material for: Defining health-related quality of life for young wheelchair users: A qualitative health economics study
Source: PLoS One. 2017 Jun 15;12(6):e0179269. doi: 10.1371/journal.pone.0179269 (PMC5472290; doi:10.1371/journal.pone.0179269)
Supplement: S2 File — A priori thematic coding framework used to code transcripts. (PDF) [file pone.0179269.s002.pdf]

Thematic coding framework

Stage 1: Descriptive themes

A. Wheelchair use

Effects of wheelchair use

- Positive impacts on life and lifestyle
- Negative impacts on life and lifestyle
- Activities (e.g. socialising, hobbies, education)

B. Quality of life and HRQoL

Defining quality of life

- Relative definition
- Relevance of outcome measures/domains

Conceptualising quality of life

- Health and quality of life
- Disability and quality of life

Impacts on quality of life

- Positive impacts on quality of life
- Negative impacts on quality of life
- Family quality of life

Measuring quality of life

- Defining domains to measure
- Generic vs. Specific

C. EQ-5D and HUI HRQoL domains

- Usual activities
- Mobility/ambulation
- Self-care
- Pain/discomfort
- Emotion/mental health
- Sight
- Hearing
- Communication
- Cognition
- Dexterity

Stage 2: Index of themes

1. **Impact of WCs on children and young people**
    1.1. Family/Parental benefits
    1.2. Lifestyle and activities
    1.3. Happiness
    1.4. Posture and comfort
    1.5. Play skills
    1.6. Functional movement and mobility
    1.7. Developmental benefits and cognition
    1.8. Safety: driving skill and competence
    1.9. Independence
    1.10. Others' perceptions
    1.11. Safety
    1.12. Access
    1.13. Self-esteem and confidence

2. **Understanding HRQoL of young WC users**
    2.1. Defining HRQoL
        2.1.1. Definition of quality of life
        2.1.2. ‘Good’ quality of life
    2.2. Conceptualising HRQoL
        2.2.1. Health and quality of life
        2.2.2. Disability and quality of life
        2.2.3. Adaptations and quality of life
        2.2.4. Disabled vs. non-disabled quality of life
    2.3. Impacts of wheelchair on HRQoL
        2.3.1. Mobility
        2.3.2. Independence and freedom
        2.3.3. Feeling ‘normal’
        2.3.4. Social interaction
        2.3.5. Health
        2.3.6. Communication
        2.3.7. Pain
        2.3.8. Happiness
        2.3.9. Adaptation
        2.3.10. Wellbeing
        2.3.11. Equality
        2.3.12. Activities and lifestyle
        2.3.13. Achievement
        2.3.14. Personal control
        2.3.15. Self-care
        2.3.16. Self-esteem and confidence
        2.3.17. Parent/family quality of life
    2.4. Measuring HRQoL
        2.4.1. Defining domains to measure
        2.4.2. Relevance of HUI domains
        2.4.3. Relevance of EQ-5D domains

Stage 3: codes, inductive categories and analytical themes

<table>
<tr><th>Pre-determined codes</th><th>Inductive categories</th><th>Analytical themes</th></tr>
<tr><td rowspan="3">Defining QoL</td><td>Activities and participation
Happiness
Independence
Social</td><td>Participation and positive experiences</td></tr>
<tr><td>Achievement and fulfilment
Being able to adapt
Emotional wellbeing
Equality
Feeling normal</td><td>Self-worth and feeling fulfilled</td></tr>
<tr><td>Cognition
Communication
Health
Mobility
Pain
Self-care</td><td>Health and functioning</td></tr>
<tr><td>Usual activities</td><td>EQ-5D-Y relevance</td><td rowspan="3">HRQoL measure relevance</td></tr>
<tr><td>Mobility/ambulation
Self-care
Pain/discomfort
Emotion/mental health</td><td>EQ-5D-Y and HUI relevance</td></tr>
<tr><td>Sight
Hearing
Communication
Cognition
Dexterity</td><td>HUI relevance</td></tr>
</table>
